# LRP5 regulates cardiomyocyte proliferation and neonatal heart regeneration by the AKT/P21 pathway

**DOI:** 10.1111/jcmm.17311

**Published:** 2022-04-16

**Authors:** Huixing Zhou, Fulei Zhang, Yahan Wu, Hongyu Liu, Ran Duan, Yuanyuan Liu, Yan Wang, Xiaoyu He, Yuemei Zhang, Xiue Ma, Yi Guan, Yi Liu, Dandan Liang, Liping Zhou, Yi‐Han Chen

**Affiliations:** ^1^ Department of Cardiology Shanghai East Hospital Tongji University School of Medicine Shanghai China; ^2^ Key Laboratory of Arrhythmias of the Ministry of Education of China Shanghai East Hospital Tongji University School of Medicine Shanghai China; ^3^ 154516 Jinzhou Medical University Liaoning Jinzhou China; ^4^ Research Units of Origin and Regulation of Heart Rhythm Chinese Academy of Medical Sciences Shanghai China; ^5^ Department of Pathology and Pathophysiology Tongji University School of Medicine Shanghai China

**Keywords:** AKT, cardiomyocyte proliferation, LRP5, neonatal heart regeneration, P21

## Abstract

The neonatal heart can efficiently regenerate within a short period after birth, whereas the adult mammalian heart has extremely limited capacity to regenerate. The molecular mechanisms underlying neonatal heart regeneration remain elusive. Here, we revealed that as a coreceptor of Wnt signalling, low‐density lipoprotein receptor‐related protein 5 (LRP5) is required for neonatal heart regeneration by regulating cardiomyocyte proliferation. The expression of LRP5 in the mouse heart gradually decreased after birth, consistent with the time window during which cardiomyocytes withdrew from the cell cycle. LRP5 downregulation reduced the proliferation of neonatal cardiomyocytes, while LRP5 overexpression promoted cardiomyocyte proliferation. The cardiac‐specific deletion of *Lrp5* disrupted myocardial regeneration after injury, exhibiting extensive fibrotic scars and cardiac dysfunction. Mechanistically, the decreased heart regeneration ability induced by LRP5 deficiency was mainly due to reduced cardiomyocyte proliferation. Further study identified AKT/P21 signalling as the key pathway accounting for the regulation of cardiomyocyte proliferation mediated by LRP5. LRP5 downregulation accelerated the degradation of AKT, leading to increased expression of the cyclin‐dependent kinase inhibitor P21. Our study revealed that LRP5 is necessary for cardiomyocyte proliferation and neonatal heart regeneration, providing a potential strategy to repair myocardial injury.

## 1 INTRODUCTION

The adult mammalian heart has limited potential for repair and regeneration.^1^ The damaged myocardium caused by ischaemic injury is replaced by scar tissue and eventually leads to heart failure. By contrast, the neonatal heart possesses a remarkable ability to regenerate following various injuries.^2^, ^3^, ^4^, ^5^ However, this regenerative potential in neonatal mammals rapidly diminishes. In mice, the regenerative capacity of neonatal hearts appears to be limited to the first 2 days after birth and basically disappears at postnatal 7 days.^6^ Emerging evidence suggests that restoration of the dormant neonatal heart regenerative ability in adulthood may provide a possible therapeutic approach for heart repair.^7^, ^8^, ^9^

Neonatal heart regeneration is orchestrated by multiple cell types and biological processes.^2^, ^10^, ^11^, ^12^, ^13^, ^14^, ^15^ Fate‐mapping studies have demonstrated that its regenerative capacity is mediated by the proliferation of pre‐existing cardiomyocytes.^2^, ^16^ Neonatal cardiomyocytes still retain unique proliferating capacity, which is lost as early as postnatal day 7 (P7d) in mice.^2^, ^16^ This timing of cell cycle withdrawal is coincident with the loss of regenerative capacity of the postnatal heart. The activation of several pathways, such as the Hippo/Yap and NRG1/Erbb2/AKT pathways,^8^, ^9^, ^17^, ^18^ has been shown to critically regulate the cell cycle re‐entry of cardiomyocytes. However, the mechanisms underlying cell cycle arrest in cardiomyocytes are largely unknown.

Low‐density lipoprotein receptor‐related protein 5 (LRP5) is a single‐span transmembrane protein known as a coreceptor in the Wnt signalling pathway.^19^ It plays an essential role in lipid metabolism, bone homeostasis and retinal vascularization.^20^, ^21^, ^22^, ^23^, ^24^, ^25^, ^26^ In addition, LRP5 is essential for the self‐renewal of hematopoietic stem cells.^27^, ^28^ Here, we revealed the critical role of LRP5 in cardiomyocyte proliferation and neonatal heart regeneration. The expression pattern of LRP5 was associated with the withdrawal of cardiomyocyte cell cycle. Cardiac‐specific knockout of *Lrp5* in neonatal mice impaired heart regeneration by inhibiting cardiomyocyte proliferation. Furthermore, we found that LRP5 deficiency accelerated the protein degradation of AKT and promoted the expression of the cyclin‐dependent kinase inhibitor P21, leading to the inhibition of cardiomyocyte proliferation.

## 2 MATERIALS AND METHODS

### 2.1 Animals

All the protocols of this study conformed to the Guidelines for the Care and Use of Laboratory Animals published by the National Institutes of Health (National Academies Press, eighth edition, 2011) and the policies of the Animal Care and Use Committee of Tongji University School of Medicine. All mice were maintained on a C57BL/6 background, and genotype was determined by PCR using tail DNA. All animal experiments were performed on age‐ and sex‐matched mice.

### 2.2 Mouse alleles and transgenic lines

Tamoxifen‐inducible cardiac‐specific knockout *Lrp5* (*Lrp5*‐CKO) mice were generated by crossing *Lrp5^flox^* ^/^ *^flox^* mice with αMHC/MerCreMer mice on a mixed C57/BL6 genetic background. DNA was extracted from the tail for genotyping. The primers for *Lrp5^flox^* ^/^ *^flox^* mice were as follows: forward, 5′‐CCACCAATCATCAGCCAAGGA‐3′ and reverse, 5′‐TCACCTGTCCTAGTGCAGAAGGA‐3′. On a 2% agarose gel, the WT allele was shown to be 166 bp, and the mutant allele generated a 288‐bp band. The αMHC‐*Cre* mice were genotyped using the following primers: 5′‐TCGATGCAACGAGTGATGAG‐3′ and 5′‐TCCATGAGTGAACGAACCTG‐3′. All animals in this study were maintained with a 12‐h cycle of light and darkness at room temperature at 22°C. To induce Cre recombinase activity, tamoxifen (T5648, Sigma) was dissolved in 90% peanut oil/10% alcohol and was administered intraperitoneally (i.p.) consecutively from P0d to P2d at a dose of 40 µg per day using an insulin needle.

### 2.3 Isolation of neonatal rat and mouse cardiomyocytes

Primary neonatal rat cardiomyocytes (NRCMs) were obtained from Sprague‐Dawley rats at P1d‐P3d. The neonatal mice cardiomyocytes (NMCMs) were obtained from P1d C57/BL6J mice. Simply, the ventricles were quickly separated and cut into small pieces (~1–3 mm^2^). Then, the tissues were digested in calcium‐free HBSS (14175095, Gibco) containing 0.125 mg/ml trypsin (15090046, Gibco), 0.1 mg/ml collagenase type IV (C4‐22‐1G, Sigma, St. Louis, Missouri, USA), and 10 mg/ml DNase II (D8764, Sigma, St. Louis, Missouri, USA). Digestion was performed by continuous stirring at 37°C, and the supernatant was collected into HBSS containing 10% FBS every 5 min. The digestion process was repeated approximately 8–10 times until the tissue was completely digested. After the supernatant was centrifuged at 1000 rpm, it was resuspended in DMEM (Gibco, Waltham, Massachusetts, USA) supplemented with 10% FBS and 100 mM 5‐bromo‐20‐deoxyuridine (B5002‐250 mg, Sigma, St. Louis, Missouri, USA). The resuspended cells were inoculated in a 10‐cm plastic dish through a cell filter (100 μm, BD Falcon, Franklin, New Jersey, USA ) at 37°C for 1.5 h to remove fibroblasts, and then, the cardiomyocytes were placed in a 1% gelatine‐coated plastic dish at an appropriate density. The culture medium was changed after 24 h of culture, and cardiac myocytes were used for subsequent cell experiments.

### 2.4 Isolation of P7d mouse cardiomyocytes

The P7d mouse cardiomyocytes were obtained from C57/BL6J mice using an established Langendorff‐free protocol with modest modification.^29^ The hearts were quickly removed, EDTA buffer (10 mM HEPES, 0.5 mM NaH_2_PO_4_, 130 mM Nalco, 5 mM KCl, 10 mM glucose, 10 mM 2, 3‐butanedione monoxime, 10 mM Taurine and 5 mM EDTA) was injected into the right ventricle to flush hearts. Then, digestion was achieved by collagenase II, IV and protease XIV. After sufficient dissociation, the digestion was stopped by 5% foetal bovine serum. Isolated cardiomyocytes were harvested for qPCR analysis and Western blot analysis.

### 2.5 Cell transfection

For siRNA knockdown experiments, Lipofectamine RNAiMAX (13778‐150, Invitrogen, USA) was used to transfect 50 nM siRNA following the manufacturer's instructions. *Lrp5* siRNA sequences are as follows: sense (5′‐CCUGGAGACUAACAACAAUTT‐3′) and antisense (5′‐AUUGUUGUUAGUCUCCAGGTT‐3′). For gene overexpression experiments, plasmids were transfected into cardiomyocytes using Lipofectamine 3000 (L3000‐008, Invitrogen，Waltham, Massachusetts, USA). Plasmids were used in the experiments: LRP5 (HG17048‐CF, Sino Biological) and AKT (39530, Addgene). The cells were harvested at 48 h after transfection.

### 2.6 Apex resection surgery

Neonatal mouse heart apex resection (AR) was performed on P1d mice, as previously described with mild modifications.^2^ P1d mice were anaesthetized by cooling on ice for 5–6 min. Following skin incision, intercostal incisions were made to separate the pericardium and expose the apex. Approximately 10% of the heart tissue was amputated with microsurgical scissors under a dissecting microscope. Following resection, the thoracic wall was then closed with 6‐0 sutures, and the skin wound was closed using skin adhesive. After surgery, the mice were warmed until recovery. Sham mice underwent the same surgical procedures without apex resection. Finally, the mice were placed back with nursing female mice. The hearts were harvested at 7 days, 14 days and 21 days post‐AR.

### 2.7 EdU assay

For NRCMs, the medium was replaced with medium containing 10 µM EdU (c10338, Thermo Fisher Scientific, component A) at 36 h after transfection, and the cells were fixed with 4% PFA 12 h later. For neonatal mice, a dose of 200 µg EdU (A10187, Thermo Fisher) per animal was injected intraperitoneally 3 days before collecting hearts. EdU incorporation into the heart sections and cells was detected with Click‐iT EdU 555 Imaging Kit reagents (c10338, Thermo Fisher Scientific, Waltham, Massachusetts, USA) according to the manufacturer's instructions. Images were obtained by Leica LAS AF software.

### 2.8 Echocardiography

Heart left ventricular systolic function was determined by serial echocardiography using Visual Sonics Vevo 770. Mice were anaesthetized with 1% isoflurane anaesthesia with spontaneous ventilation. Ejection fraction and fractional shortening were calculated based on end‐diastolic and end‐systolic dimensions obtained from M‐mode ultrasound. All echocardiography measurements were performed in a blind manner.

### 2.9 Histology

For histological analysis, hearts were collected and fixed with 4% paraformaldehyde (PFA, Sigma, St. Louis, Missouri, USA) at 4°C overnight, dehydrated, embedded in paraffin and sectioned frontally at 6 µm thickness. Haematoxylin and eosin staining and Masson's trichrome staining were performed according to previously published methods. H&E staining was performed to detect cardiac structure defects. Masson's trichrome staining was performed to detect cardiac fibrosis and infarction size, respectively. Infarction size was quantified using the ImageJ software.

### 2.10 Immunofluorescence

The cultured cardiomyocytes were fixed with 4% PFA for 15 min at time temperature. Samples were washed twice with PBS followed by 0.5% Triton X‐100 in PBS incubation for 10 min. Then, cells were blocked with 4% normal goat serum for 1h at time temperature. Then, the cells were incubated with primary antibodies overnight at 4°C. Following three washes in PBST, the samples were incubated with appropriate secondary antibodies for 1 h at time temperature followed by 10 min of DAPI staining to label nuclei.

For paraffin‐embedded heart tissues, heart slides were deparaffinized with xylene and rehydrated with descending concentrations of ethanol, followed by microwave antigen retrieval in citrate solution for 10 min. The slides were incubated with primary antibodies overnight at 4°C after nonspecific sites were blocked with 10% goat serum (Invitrogen, Waltham, Massachusetts, USA). The next day, after being washed with PBST three times, the slides were incubated with appropriate secondary antibodies for 1 h at time temperature, washed in PBST again and then stained with DAPI for 10 min to label nuclei. Images were processed by using Leica LAS AF software. All immunostaining was performed on three discontinuous sections per heart, and 3–10 hearts of each group were tested. Images were acquired for 5 fields per section, processed and quantified using the ImageJ software.

The primary antibodies for immunofluorescence in this study included mouse anti‐cTnT (1:100, ab8259, Abcam, Cambridge, United Kingdom) and rabbit anti‐PCM1 (1:100, HPA023370 Sigma, St. Louis, Missouri, USA ), which was used to identify cardiomyocytes in cultured cells and heart tissue slides. Anti‐phosphorylated histone H3 (1:500, ab32107, Abcam, Cambridge, United Kingdom or 1:500, 9706 S, CST, Danvers, Massachusetts, USA) and Anti‐Ki67 (1:500, ab16667, Abcam,Cambridge, United Kingdom ) were used to analyse cell cycle activity. The isolectin B4 antibody (B‐1205, Vector Labs, Burlingame, California, USA) was used to examine vessel density. For wheat germ agglutinin (WGA) staining, the heart sections were deparaffinized, rehydrated and then incubated for 30 min at room temperature with WGA conjugated to Alexa Fluor‐594 (50 mg/ml, W11262, Thermo Fisher Scientific, Waltham, Massachusetts, USA) in PBS. The cell size was quantified manually using the ImageJ software. Anti‐LRP5 (1:50, ab36121, Abcam, Cambridge, United Kingdom) and anti‐AKT (1:50, sc‐81434, Santa Cruz Biotechnology, Dallas, Texas, USA) were used to detect the co‐localization of LRP5 and AKT in NRCMs. The secondary antibodies used in this study were as follows: conjugated to Alexa Fluor‐488 or Alexa Fluor‐555 (1:200, Abcam, Cambridge, United Kingdom). The observer was blinded to genotypes and measurements.

### 2.11 Quantitative real‐time PCR

Total RNA was isolated from heart tissues and cultured CMs using TRIzol reagent (Invitrogen) according to the manufacturer's protocol. Total RNA was reverse transcribed to cDNA using qScript cDNA SuperMix (RR036A, Takara, Kusatsu, Shiga, Japan). qPCR was performed on a StepOnePlus Real‐Time PCR System (Applied Biosystems) using SYBR Green Supermix (A25742, Applied Biosystems, Waltham, Massachusetts, USA ). The relative mRNA expression of each target gene was normalized to the *GAPDH* housekeeping control and calculated using the 2^−ΔΔCT^ method. Biological replicates were performed using three individual samples of each genotype. The specific primers for qPCR are listed in Table S1.

### 2.12 Western blot analysis

Western blotting was performed with the SDS‐PAGE electrophoresis system. Total proteins of heart tissues and cultured CMs were extracted in cold RIPA lysis buffer plus protease inhibitor (Roche, Basel, Switzerland). Protein concentration was quantified using a Pierce BCA protein assay kit (P0009, Beyotime, Shanghai, China). Equal amounts of protein (70 µg for heart tissues, 50 µg for cells) were mixed and resolved in 4 × SDS‐PAGE sample buffer and boiled for 5 min at 95°C. After separation via NuPAGE 10% Bis‐Tris Gels (Invitrogen, Waltham, Massachusetts, USA), proteins were transferred to PVDF membranes (EMD Millipore, Burlington, Massachusetts, USA). Membranes were blocked in 5% milk/TBS‐Tween 20 for 30 min and incubated with appropriate primary antibodies overnight at 4°C. A conjugated fluorescent secondary antibody (Li‐Cor) was used as the secondary antibody. Protein detection was performed by using an Odyssey imager. For quantification, band densities were determined using the ImageJ software (National Institutes of Health).

Proteins were detected with the following primary antibodies: LRP5 (5731S, Cell Signaling Technology, Danvers, Massachusetts, USA), P21 (ab109199, Abcam, Cambridge, United Kingdom), P27 (sc‐1641, Santa Cruz Biotechnology, Dallas, Texas, USA), phospho‐P27 (ab62364, Abcam, Cambridge, United Kingdom), P53 (ab26, Abcam, Cambridge, United Kingdom), P38 (ab170099, Abcam, Cambridge, United Kingdom), phospho‐P38‐Thr180/Tyr182 (4511, Cell Signaling Technology, Danvers, Massachusetts, USA), AKT (sc‐81434, Santa Cruz Biotechnology, Dallas, Texas, USA), phospho‐AKT‐Ser (sc‐514032, Santa Cruz Biotechnology, Dallas, Texas, USA), phospho‐AKT‐Thr (sc‐271966, Santa Cruz Biotechnology, Dallas, Texas, USA), mTOR (ab32028, Abcam, Cambridge, United Kingdom), PI3K (4257, Cell Signaling Technology, Danvers, Massachusetts, USA ) and ubiquitin (ab134953, Abcam, Cambridge, United Kingdom).

### 2.13 Co‐Immunoprecipitation

Cultured cardiomyocytes were homogenized and lysed in a buffer containing RIPA buffer (Beyotime) and protease inhibitor cocktail (Roche, Basel, Switzerland). Total cell lysates were centrifuged at 14,000 × g for 15 min at 4°C, and the supernatant was collected for further lysis. Then, 30 μl Protein A agarose (P2009, Beyotime, Shanghai, China) beads were added and shaken at 4°C for 4 h to remove nonspecific complex proteins. Anti‐LRP5 (2 µg, 5731S, Cell Signaling Technology, Danvers, Massachusetts, USA ), anti‐AKT (2 µg, ab8805, Abcam, Cambridge, United Kingdom) and normal rabbit IgG (2 µg, 03‐241, Millipore, Burlington, Massachusetts, USA), which was used as a negative control, were added to lysates and incubated overnight. The next day, 30 μl Protein A agarose beads were added to capture the antigen–antibody complex at 4°C for 4 h, and the immunoprecipitates were subjected to Western blotting as described above.

### 2.14 Protein stability study

NRCMs were incubated with cycloheximide (CHX) (5 µM, A8244, ApexBio, Houston, Texas, USA) after 48 h of transfection. Whole‐cell proteins were harvested at 0 h, 6 h, 12 h and 24 h after treatment. Protein lysates were used for immunoblotting as described.

### 2.15 Protein synthesis study

The cardiomyocytes were treated with 5 µM puromycin (CL13900) 2HCl (S7417, Selleck Chemicals, Houston, Texas, USA) for 1 h before collection. Whole‐cell protein lysates were extracted for Western blot analysis. Anti‐puromycin (EQ0001, Kerafast, Boston, Massachusetts, USA) was used as the primary antibody.

### 2.16 Proteasome inhibition study

Cardiomyocytes were co‐incubated with 4 µM MG132 (S2619, Selleck Chemicals, Houston, Texas, USA) or DMSO for 24 h. Then, the cellular protein was harvested and further processed for Western blotting.

### 2.17 Lysosome inhibition study

Cardiomyocytes were treated with 1 µM (S)‐Hydroxychloroquine (HY‐B1370A, MCE, Monmouth Junction, New Jersey, USA ) or DMSO for 24 h. Then, the NRCMs were collected for Western blot analysis.

### 2.18 Statistical analysis

Results are presented as the mean ± SD. Statistical analysis was performed with GraphPad Prism software 8.0. Differential expression analysis was analysed with unpaired Student's *t*‐tests (two‐tailed), and one‐way ANOVA followed by Tukey's post hoc test for two or multiple groups, respectively. *p* < 0.05 was considered to be statistically significant.

## 3 RESULTS

### 3.1 LRP5 is required for cardiomyocytes proliferation

**FIGURE 1:**
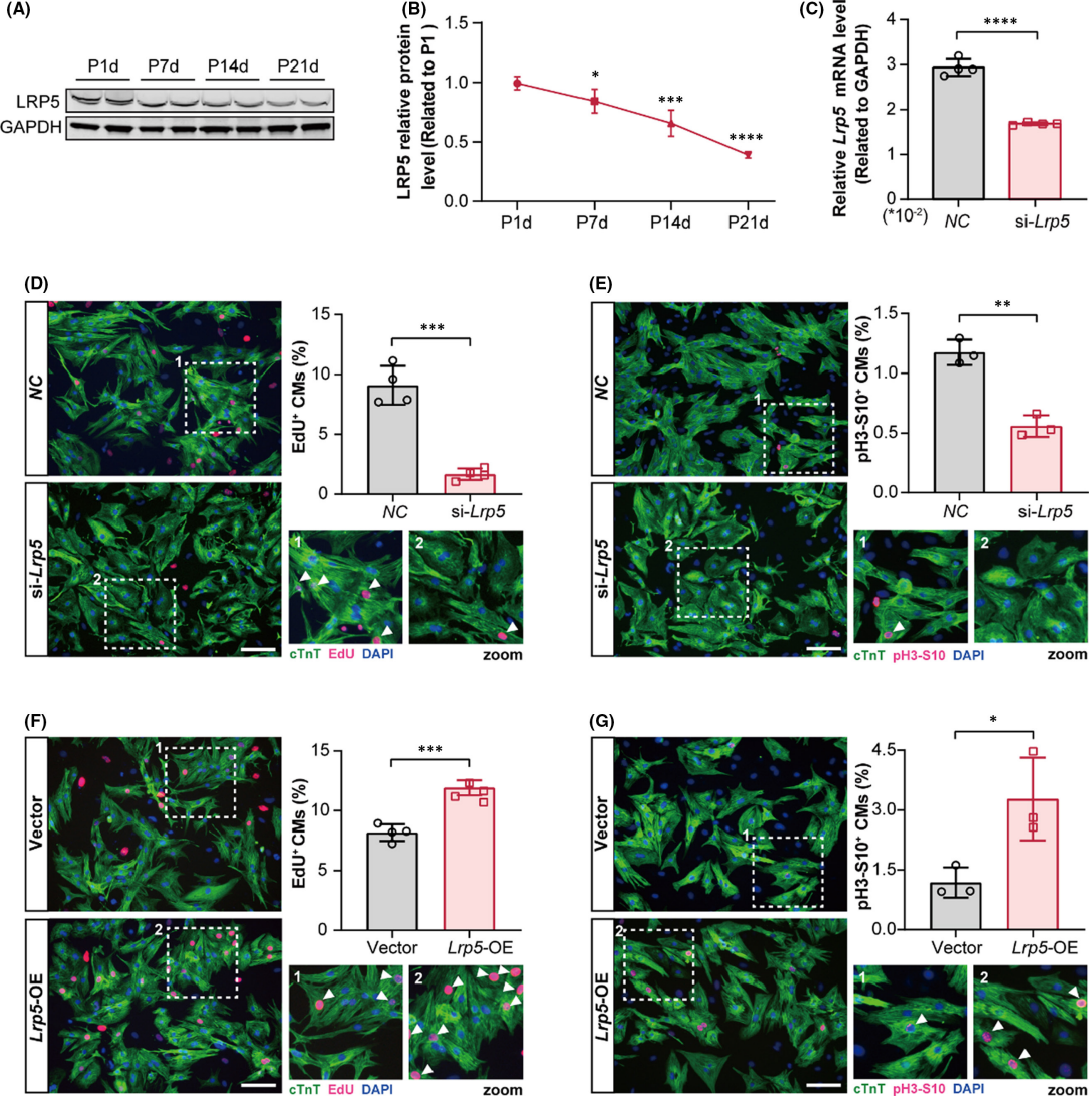
LRP5 is required for neonatal cardiomyocyte proliferation. (A, B) Western blot shows the expression of LRP5 in mouse heart tissues after birth. Wild type mouse hearts from 1 day, 7 days, 14 days and 21 days (P1d, P7d, P14d, P21d) after birth were harvested for LRP5 expression examination. *n* = 4–6 mice per group. *p < 0.05, **p < 0.01, ***p < 0.001, ****p < 0.0001 versus P1d heart tissues. Data were presented as means ± SD. (C) The knockdown efficiency of *Lrp5* siRNA in rat neonatal cardiomyocytes (NRCMs) using qPCR assay. NRCMs were transfected with negative control (*NC*) siRNA and *Lrp5* siRNA for 48 h. Four independent experiments were performed. The data are presented as the means ± SD. ****p < 0.0001. EdU staining and pH3‐S10 staining were used to evaluate the proliferative ability of cardiomyocytes in (D–G). (D) Evaluation of NRCMs proliferative activity by EdU staining following *Lrp5* knockdown. NRCMs were treated with EdU (10 µM) at 36 h after transfection, and the cells were fixed 12 h later. Fluorescence was observed by using microscopy. Representative images show the NRCMs staining with EdU and cTnT in *NC* or si‐*Lrp5* group. White arrows indicate EdU^+^cTnT^+^ cells. The column showing the quantification of percentage of EdU^+^ cardiomyocytes. Scale bar, 100 µm. Values are the average ± SD of three independent experiments. *p* values were calculated using the unpaired Student's *t* test (***p < 0.001). (E) Evaluation of NRCMs proliferative activity by staining pH3‐S10 following *Lrp5* knockdown. Representative images of NRCMs staining with pH3‐S10 and cTnT in *NC* or si‐*Lrp5* group. White arrows indicate pH3‐S10^+^cTnT^+^ cells. The column showing the quantification of percentage of pH3‐S10^+^ cardiomyocytes. Scale bar, 100 µm. Values are the average ± SD of 4 independent experiments. *p* values were calculated using the unpaired Student's *t* test (**p < 0.01). (F, G) Evaluation of cardiomyocyte proliferative activity by EdU staining (F) or pH3‐S10 staining (G) following LRP5 overexpression (*Lrp5*‐OE). NRCMs were treated with vehicle plasmid and *Lrp5* overexpressing plasmid respectively for 48 h before fixation and staining. Representative images showing the EdU‐positive or pH3‐S10‐positive cardiomyocytes after LRP5 overexpression, respectively. White arrows indicate EdU^+^cTnT^+^ cells or pH3‐S10^+^cTnT^+^ cells, respectively. Scale bar, 100 µm. Values are the average ± SD of three (G) or four (F) independent experiments. *p* values were calculated using the unpaired Student's *t* test (*p < 0.5, ***p < 0.001)

To clarify the potential role of LRP5 in cardiomyocyte proliferation, we first examined the expression of LRP5 in hearts during postnatal development. The protein expression of LRP5 was detected in mouse cardiac tissues on P1d, P7d, P14d and P21d. A gradual decrease in LRP5 expression was observed from P7d (Figure 1A and B), which coincided with the proliferative time window of cardiomyocytes.^2^, ^6^ We further examined the protein expression of LRP5 in neonatal mouse cardiomyocytes (NMCMs) on P1d and P7d to confirm its reduction. The expression of LRP5 was significantly downregulated in P7d cardiomyocytes (CMs) when compared with P1d CMs (Figure S1). We then performed siRNA‐mediated *Lrp5* knockdown experiments in NRCMs (Figure 1C) to examine the effect of LRP5 downregulation on cardiomyocyte proliferation. By 5‐ethynyl‐2'‐deoxyuridine (EdU, a DNA synthesis marker), phospho‐Histone H3‐Ser10 (pH3‐S10, a mitosis marker) and Ki67 (a cell proliferative marker) staining, we found that LRP5 downregulation resulted in 82% decrease of EdU‐positive cardiomyocytes (Figure 1D), 48% decrease of pH3‐S10‐positive cardiomyocytes (Figure 1E), as well as 42% decrease of Ki67‐positive cardiomyocytes (Figure S2). To investigate whether LRP5 overexpression could promote cardiomyocyte proliferation, we transfected an *Lrp5* plasmid into NRCMs (Figure S3). The results showed that LRP5 overexpression significantly increased the percentages of EdU‐positive (~50%), pH3‐S10‐positive (~1.7 fold) and Ki67‐positive (~62%) cardiomyocytes compared to controls (Figure 1F and G, Figure S4). These data demonstrated that LRP5 could regulate cardiomyocyte proliferation *in vitro*.

### 3.2 Cardiac‐specific conditional knockout of *Lrp5* inhibits cardiomyocyte proliferation, interrupting neonatal heart regeneration

**FIGURE 2:**
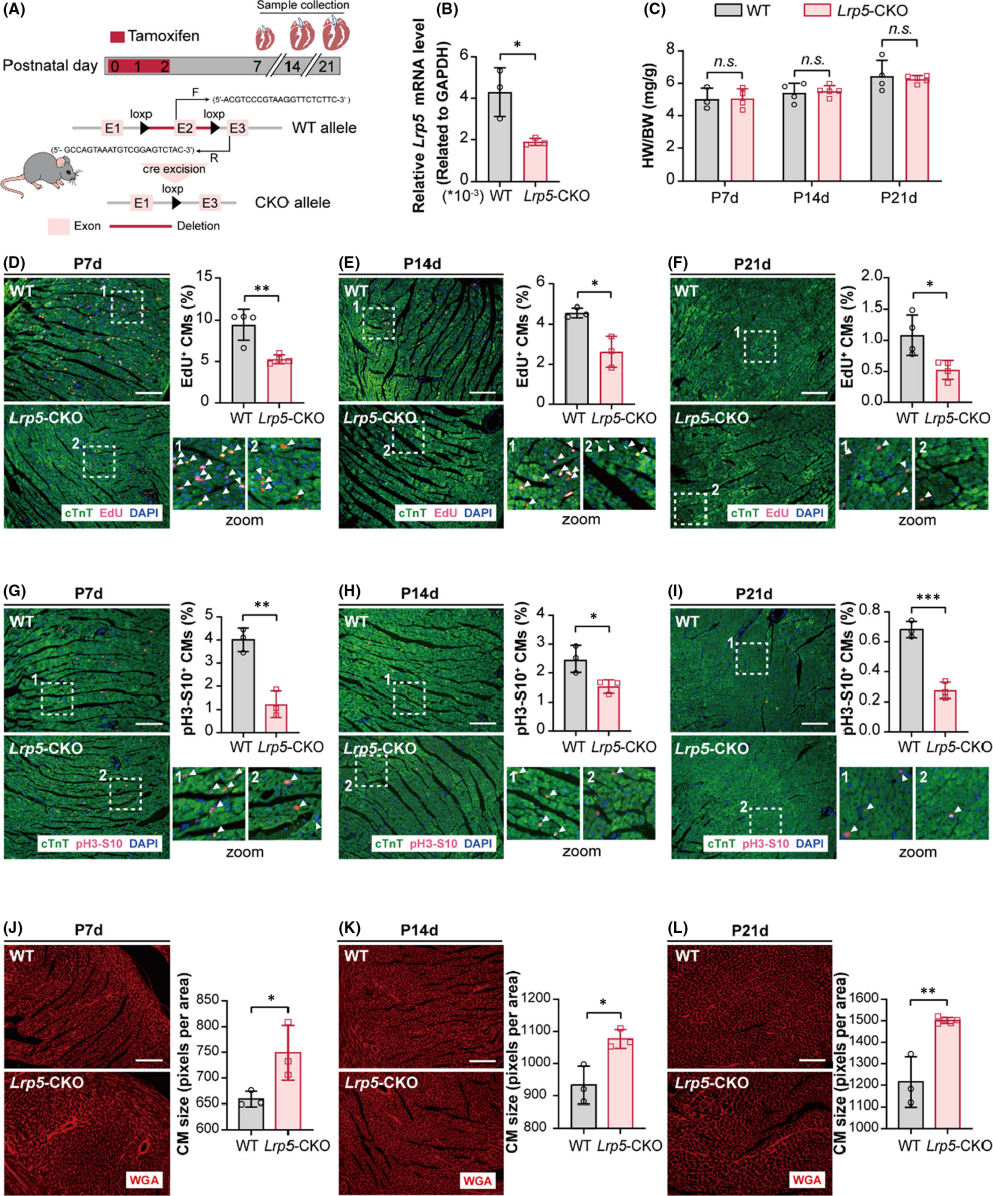
Cardiac‐specific knockout of *Lrp5* inhibits cardiomyocyte proliferation. (A) *Top*, experimental timeline; *bottom*, a schematic diagram of the construction of cardiomyocyte‐specific knockout mice. (B) qPCR assay demonstrates effective deletion of *Lrp5* in cardiac‐specific knockout of *Lrp5* (*Lrp5*‐CKO) mouse heart tissues. The neonatal mice (P0d) were injected with tamoxifen (40 ug) for 3 days, the total RNA extracted from P3d littermate control (WT) and *Lrp5*‐CKO mouse hearts for efficiency of *Lrp5* deletion examination. Values are the average ± SD of three mice per group. *p < 0.05. (C) Measurement of the heart weight (HW) to body weight (BW) ratio in WT and *Lrp*5‐CKO mice at P7d, P14d and P21d. *n* = 3–5 mice per group. The data are presented as the means ± SD. *n*.*s*., represents not significance. (D–F) EdU staining showing the cardiomyocyte proliferative activity of WT and *Lrp5*‐CKO mice at P7d, P14d and P21d, respectively. WT and *Lrp5*‐CKO mice were administrated intraperitoneally with EdU (200 µg) at 3 days before hearts collected. The representative images showing the heart slides from WT and *Lrp5*‐CKO hearts stained with EdU and cTnT at P7d, P14d and P21d, respectively. Graph showing the percentages of EdU^+^ cardiomyocytes in WT and *Lrp5*‐CKO mouse hearts slides at P7d, P14d and P21d, respectively. Scale bar, 100 µm. *n* = 3–4 mice per group, and 3 discontinuous whole heart sections per sample were counted. The data are presented as the means ± SD. *p < 0.05, **p < 0.01 versus WT group. (G–I) pH3‐S10 staining showing the cardiomyocyte proliferative activity in WT and *Lrp5*‐CKO mice at P7d, P14d and P21d. The representative images showing the heart slides from WT and *Lrp5*‐CKO mice hearts staining with pH3‐S10 and cTnT at P7d, P14d and P21d, respectively. Graph showing the percentages of pH3‐S10^+^cTnT^+^ cells in WT and *Lrp5*‐CKO mice slides at P7d, P14d and P21d, respectively. Scale bar, 100 µm. *n* = 3 mice per group, and 3 discontinuous whole heart sections per sample were counted. The data are presented as the means ± SD. *p < 0.05, **p < 0.01, ***p < 0.001. (J–L) WGA staining showing cardiomyocyte size in WT and *Lrp*5‐CKO mice at P7d, P14d and P21d. Representative images showing the WGA staining of the heart slides from WT and *Lrp*5‐CKO mice at P7d, P14d and P21d, respectively. Graph showing the quantification of transverse cell size in WT and *Lrp*5‐CKO mouse hearts at P7d, P14d, P21d. Scale bar, 100 µm. *n* = 3–5 mice per group, approximately 1000 cells per mouse were counted. Scale bar, 100 µm. The data are presented as the means ± SD.*p < 0.05, **p < 0.01, versus WT group

To confirm the effect of LRP5 on cardiomyocyte proliferation *in vivo*, we established cardiac conditional deletion mice by crossing *Lrp5^flox^* ^/^ *^flox^* mice with α*MHC*/MerCreMer mice (Figure 2A). Neonates were given tamoxifen at birth for three days. The effective deletion of LRP5 in hearts was confirmed by qPCR and Western blot (Figure 2B, Figure S5). We found that there was no difference in the ratio of heart weight and body weight between WT (littermate control) and cardiac‐specific conditional knockout of *Lrp5* (*Lrp5*‐CKO) mice (Figure 2C). No differences were observed in myocardial fibrosis examined by Masson's staining (Figure S6A) or in heart size examined by H&E staining between WT and *Lrp5*‐CKO mice (Figure S6B). Immunofluorescence analysis of heart tissue slides showed that the *Lrp5*‐CKO hearts displayed significantly decreased proliferation of cardiomyocytes evaluated by reduced percentage of EdU‐ and pH3‐S10‐positive cardiomyocytes compared to WT mice at P7d, P14d and P21d (EdU: P7d, decreased by 44%, P14d, decreased by 42%, P21d, decreased by 52%; pH3‐S10, P7d, decreased by 69%, P14d, decreased by 39%, P21d and decreased by 60%) (Figure 2D‐I). In addition, WGA staining showed that the cell size was increased in *Lrp5*‐CKO mice at P7d, P14d and P21d (Figure 2J‐L), which may be the reason why *Lrp5*‐CKO mice had decreased cardiomyocyte proliferation but had no significant differences in heart weight ratios compared with WT mice. Collectively, our results showed that LRP5 was necessary for cardiomyocyte proliferation in neonatal hearts.

**FIGURE 3:**
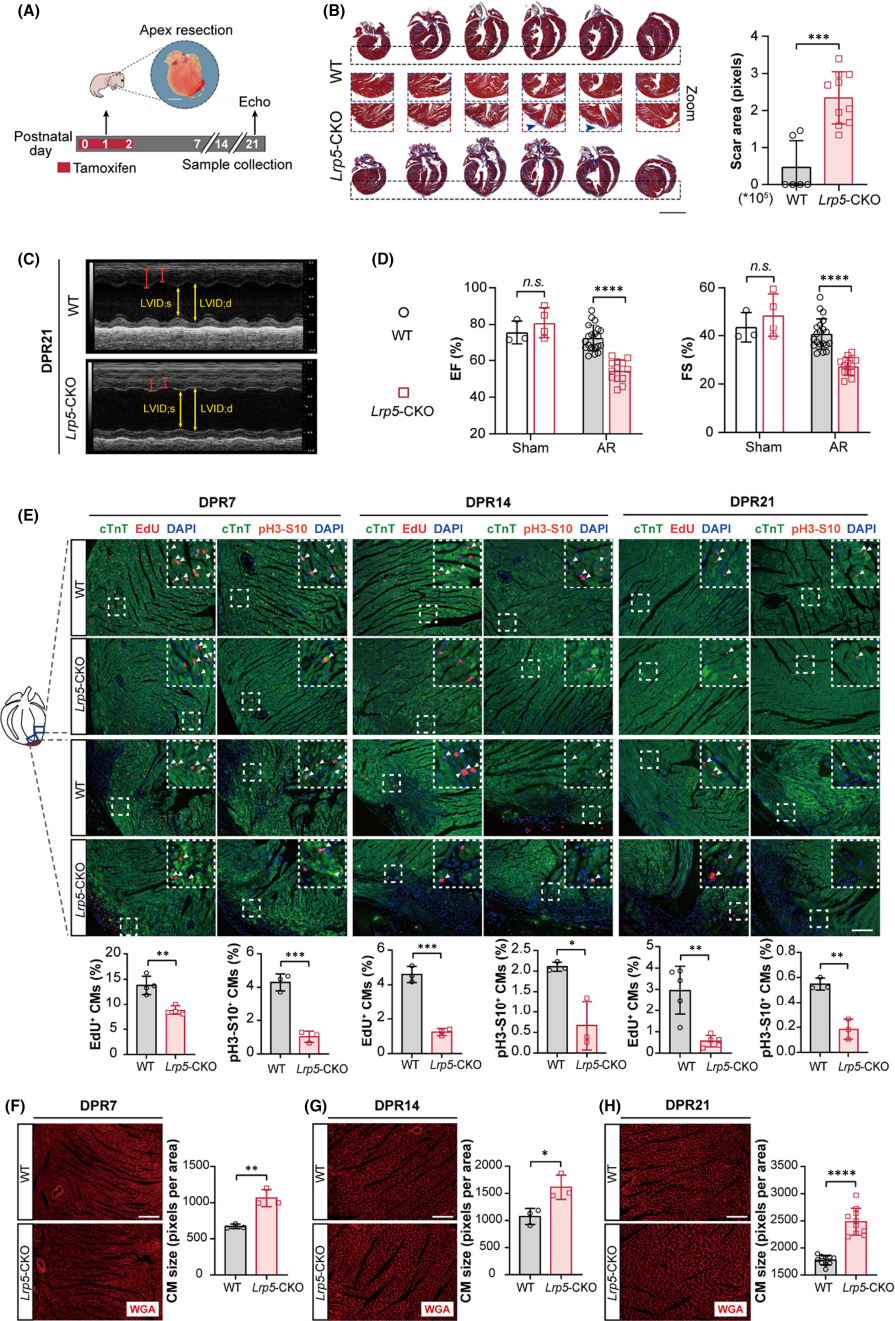
LRP5 deficiency impairs neonatal heart regeneration. (A) Experimental timeline. Echo: echocardiography. Littermate control (WT) and *Lrp5*‐CKO neonatal mice (P1d) were performed apex resection (AR) surgery. The hearts of WT and *Lrp5*‐CKO mice were harvested at 7 days, 14 days and 21 days post apex resection (DPR7, DPR14 and DPR21) for Masson's trichrome staining and immunofluorescence staining. The echo was performed at DRP21. Scale bar, 1 mm. (B) Representative images of Masson's trichrome staining (*left*) and scar size quantification (*right*) of WT and *Lrp5*‐CKO mice at DPR21. The blue arrow represents fibrosis at the apex of the heart. Scale bar, 2 mm. *n* = 6–10 mice/group. The data are presented as the means ± SD. ***p < 0.001. (C) Representative images of M‐mode echocardiography of WT and *Lrp5*‐CKO mice at DPR21. (D) Graph showing left ventricular systolic function evaluated by ejection fraction (EF%) and shortens fraction (FS%) of WT and *Lrp5*‐CKO mice at DPR21. For sham surgery group, *n* = 3–4 mice/group, for apex resection (AR) surgery group, *n* = 13–20 mice/group. Data are presented as the means ± SD. *n*.*s*., represents no significance, **** represents *p* < 0.0001. (E) Immunofluorescence analysis of EdU and pH3‐S10 in apical sections of WT and *Lrp5*‐CKO hearts at DPR7, DPR14 and DPR21. *Top*, representative images of EdU or pH3‐S10 staining in apical sections of WT and *Lrp5*‐CKO mice at DPR7, DPR14, and DPR21, *bottom*, quantification of EdU‐ or pH3‐S10‐positive cardiomyocytes in apical sections of WT and *Lrp5*‐CKO mice at DPR7, DPR14 and DPR21. Scale bar, 100 µm, *n* = 3–5 mice/group. Data are presented as the means ± SD. *p < 0.05, **p < 0.01, ***p < 0.001. (F–H) Representative images of WGA staining (*left*) and quantification of cell size (*right*) in WT and *Lrp5*‐CKO hearts at DPR7, DPR14 and DPR21. Scale bar, 100 µm. *n* = 3–10 mice per group, approximately 1000 cells were quantified per mouse. The data are presented as the means ± SD.*p < 0.05, **p < 0.01, ****p < 0.0001

We then examined whether cardiac regeneration was affected in *Lrp5*‐CKO neonatal mice using an apex resection (AR) model (Figure 3A). We found that the injured hearts of WT mice could regenerate as expected at 21 days post‐AR (DPR21), while a large fibrotic scar area was observed in the apex of *Lrp5*‐CKO hearts at DPR7, DPR14 and DPR21, as shown by Masson's trichrome staining and scar size quantification (Figure 3B, Figure S7). Notably, consistent with the scar phenotype, *Lrp5*‐CKO mice exhibited an obvious reduction in heart function compared to WT mice at DPR21, as determined by echocardiography (EF%, decreased by 25%; FS%, decreased by 33%) (Figure 3C and D, Figure S8). Further immunofluorescence staining of EdU and pH3‐S10 revealed that cardiomyocyte proliferation was reduced in the injured apical sections of *Lrp5*‐CKO mice at DPR7, DPR14 and DPR21 (EdU: DPR7, decreased by 36%, DPR14, decreased by 73%, DPR21, decreased by 81%; pH3‐S10, DPR7, decreased by 76%, DPR14, decreased by 68%, DPR21, decreased by 66%) (Figure 3E), respectively. In addition, enlarged cardiomyocytes were also identified in *Lrp5*‐CKO mice at DPR7, DPR14 and DPR21 (Figure 3F–H).

Considering that angiogenesis is involved in neonatal mouse regeneration,^30^ we then performed IB4 staining to detect the vascular density in WT and *Lrp5*‐CKO hearts at DPR21. There were no differences in vascular density between WT and *Lrp5*‐CKO hearts (Figure S9). Above all, these results indicated that the impaired ability of neonatal heart regeneration induced by LRP5 deficiency was mainly due to reduced cardiomyocyte proliferation rather than angiogenesis.

### 3.3 LRP5 regulates cardiomyocyte proliferation via the AKT pathway

As a well‐known coreceptor, LRP5 conducts Wnt signals through several cytoplasmic relay elements to effect cytoplasmic stabilization of β‐catenin and eventually initiates typical cascade signals.^19^ Our previous study showed that LRP5 deletion did not alter the total protein expression of Axin2, GSK‐3β or β‐catenin, which were critical downstream components of Wnt signalling. Importantly, LRP5 deletion did not alter β‐catenin protein expression in either the nucleus or cytoplasm.^31^ To further investigate whether LRP5 regulated cardiomyocyte proliferation *via* the Wnt signalling pathway, we added Wnt3a (an agonist of the Wnt signalling pathway) to LRP5‐deficient NRCMs and performed EdU assay. We found that Wnt3a could not attenuate the decrease of cardiomyocyte proliferation caused by LRP5 deficiency (Figure S10). These data suggested that the effect of LRP5 on cardiomyocyte proliferation did not depend on the Wnt/β‐catenin signalling pathway.

**FIGURE 4:**
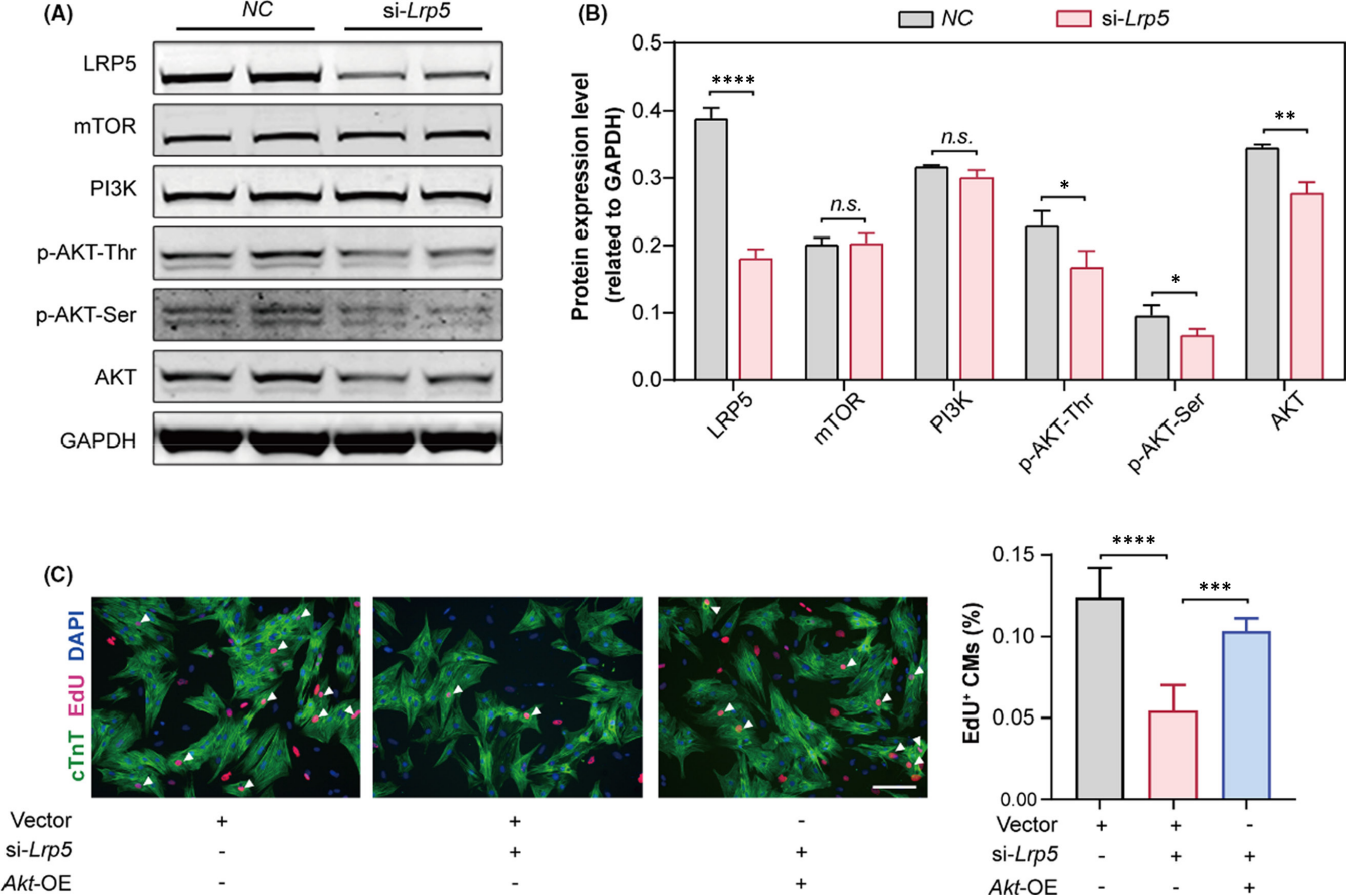
LRP5 regulates cardiomyocyte proliferation via the AKT pathway. (A, B) Western blot analysis showing the protein expression of total AKT, phospho‐AKT, PI3K and mTOR in negative control (*NC*) and *Lrp5*‐siRNA‐treated NRCMs (*si*‐*Lrp5*). NRCMs were transfected with *NC*‐siRNA or *Lrp5* siRNA for 48 h, and then, the total proteins were extracted for detection. (A) Representative Western blot bands, (B) pooled data. GAPDH was used as a loading control. *n* = 3 independent experiments. The data are presented as the means ± SD. *n*.*s*., not significant, *p < 0.05, **p < 0.01, ****p < 0.0001. (C) EdU assays showing that the decreased proliferation of cardiomyocytes induced by LRP5 deficiency was attenuated by AKT overexpression. NRCMs were transfected with vector plasmid, *Lrp5*‐siRNA + vector plasmid or *Lrp5*‐siRNA + Akt overexpressed plasmid for 48 h, respectively. *Left*, representative images showing EdU staining; *right*, quantification of EdU^+^cTNT^+^ cells. Scale bar, 100 µm. *n* = 5 independent experiments. The data are presented as the means ± SD. ****p < 0.0001, versus vector group, ***p < 0.001, versus *Lrp5*‐siRNA‐treated group

PI3K‐AKT signalling is an important pathway tightly associated with the control of cardiomyocyte proliferation.^32^, ^33^ Previous studies have found that the LRP5‐AKT pathway is involved in the self‐renewal of hematopoietic stem cells.^27^ To investigate whether AKT is a key downstream target of LRP5 in the regulation of cardiomyocyte proliferation, we first examined AKT expression after LRP5 knockdown. The result showed that the protein expression of total AKT and phosphorylated AKT was robustly decreased in LRP5‐downregulated NRCMs (Figure 4A and B). In addition, we found that the protein expression of PI3K and mTOR, important components involved in the PI3K‐AKT pathway, was not changed in NRCMs after LRP5 knockdown (Figure 4A and B), suggesting that the reduction in AKT protein expression after LRP5 downregulation is not due to inhibition of the classic PI3K‐AKT pathway.

To further determine whether the reduction in AKT protein expression was responsible for the decreased cardiomyocytes proliferation induced by LRP5 knockdown, we next examined cardiomyocyte proliferation after AKT overexpression using an EdU assay. We found that AKT overexpression attenuated the decrease in cardiomyocyte proliferation induced by LRP5 deficiency (Figure 4C, Figure S11). Collectively, these results demonstrated that AKT mediated the effect of LRP5 on cardiomyocyte proliferation.

### 3.4 LRP5 deficiency accelerates the protein degradation of AKT

**FIGURE 5:**
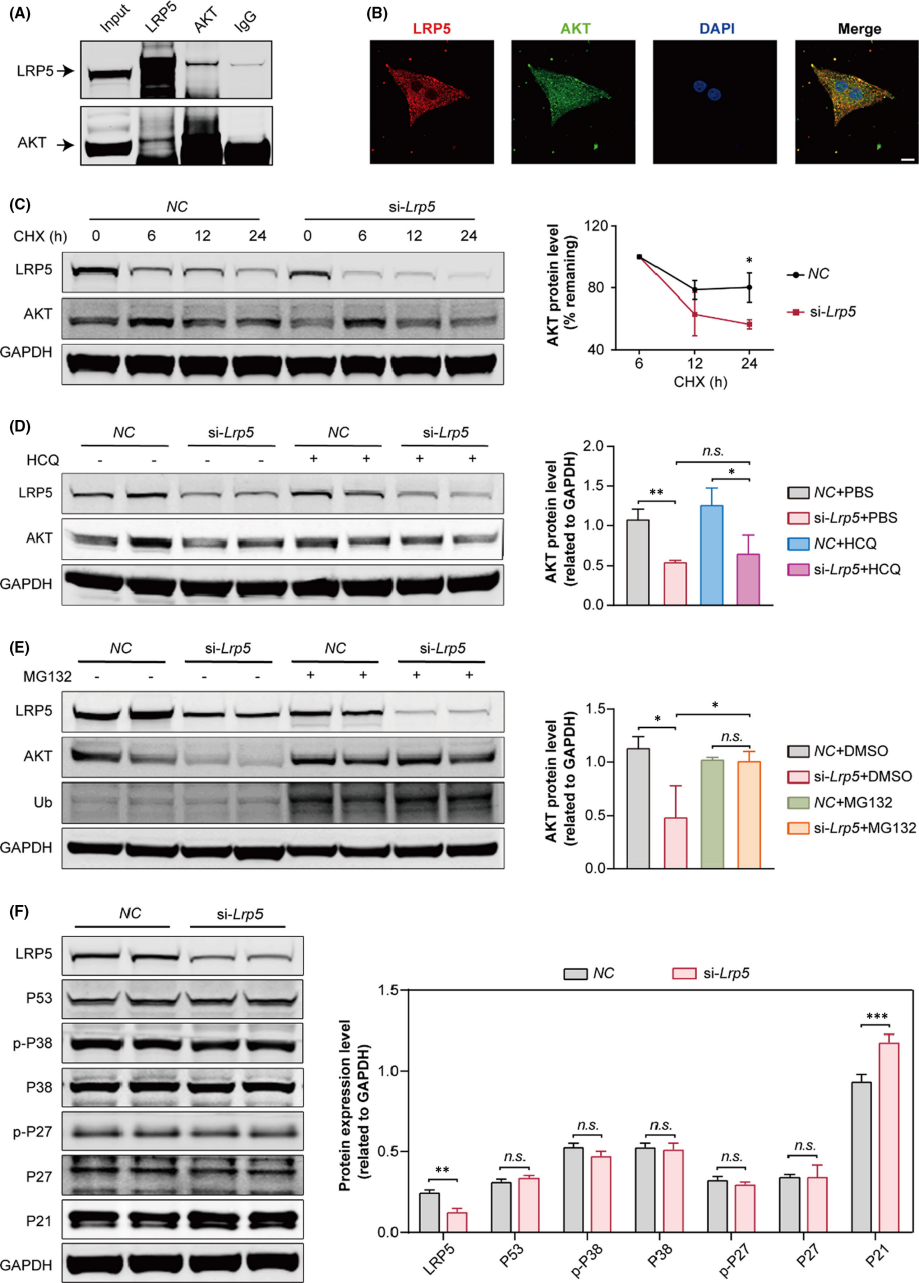
LRP5 deficiency accelerates AKT degradation. (A) Coimmunoprecipitation of endogenous LRP5 and AKT in NRCMs. (B) Representative images showing the co‐localization of LRP5 and AKT in NRCMs. Scale bar, 10 µm. (C) NRCMs were transfected with negative control (*NC*) siRNA or *Lrp5* siRNA for 48 h, respectively, then NRCMs were treated with cycloheximide (CHX) (5 µM) for 0 h, 6 h, 12 h and 24 h. The cells were collected at indicated time for AKT protein stability assay. *Left*, representative Western blot bands showing the stability of total AKT following *Lrp5* knockdown in NRCMs. *Right*, time course showing the loss of total AKT after LRP5 knockdown in NRCMs. GAPDH was used as a loading control. *n* = 3 independent experiments. The data are presented as the means ± SD. *p < 0.05 versus *NC* group. (D) The effect of Hydroxychloroquine (HCQ, lysosomal inhibitor) on AKT protein expression in *Lrp5* siRNA‐treated NRCMs. NRCMs were transfected with NC‐siRNA or *Lrp5* siRNA for 24 h and then were treated with PBS or HCQ (1 µM) for 24 h, respectively. The NRCMs were collected for western blot analysis. *Left*, representative Western blot bands; *right*, pooled data. GAPDH was used as a loading control. *n* = 3 independent experiments. The data are presented as the means ± SD. *n*.*s*., represents not significant, * represents *p* < 0.05. (E) The effect of proteasome inhibition (MG132) on AKT protein expression and AKT protein ubiquitination in *Lrp5* siRNA‐treated NRCMs. NRCMs were transfected with *NC*‐siRNA or *Lrp5* siRNA for 48 h, and then, cells were treated with DMSO or MG132 (4 µM) for 24 h, respectively. The NRCMs were collected for Western blot analysis. *Left*, representative blot bands; *right*, pooled data. GAPDH was used as a loading control. *n* = 3 independent experiments. The data are presented as the means ± SD. *n*.*s*., represents not significant, * represents *p* < 0.05. (F) The P21, P27, p‐P27, P38, p‐P38 and P53 protein expression levels were examined in *NC* and *Lrp5* siRNA‐treated NRCMs. The column shows the quantification of P21, P27, p‐P27, P38, p‐P38 and P53 protein expression relative to GAPDH in *NC* and *Lrp5* siRNA‐treated NRCMs. *n* = 3 independent experiments. The data are presented as the means ± SD. *n*.*s*., not significant, **p < 0.01, ***p < 0.001

To investigate the potential molecular mechanism underlying the reduction in AKT protein levels after LRP5 deficiency, we first examined the mRNA level of *Akt* in LRP5‐deficient NRCMs, which was not changed (Figure S12). To assess the possible interaction between LRP5 and AKT, we performed coimmunoprecipitation experiments in NRCMs and found that LRP5 could directly bind to AKT (Figure 5A). To further clarify the binding of LRP5 and AKT, we performed co‐immunofluorescence staining for LRP5 and AKT in NRCMs. The results showed that LRP5 and AKT were co‐localized in NRCMs (Figure 5B).

To examine whether LRP5 deficiency affected AKT protein synthesis, we treated NRCMs with puromycin (a protein synthesis inhibitor, for monitoring new protein synthesis) and then detected its level after LRP5 knockdown. We found that the level of puromycin was not changed after LRP5 downregulation, suggesting that LRP5 deficiency did not affect nascent protein synthesis (Figure S13). To determine whether LRP5 deficiency affected the protein degradation of AKT, we treated control and LRP5‐deficient NRCMs with cycloheximide (CHX) (a protein synthesis inhibitor, for detection of protein half‐life). The AKT protein was upregulated at 6 h in both control and LRP5‐deficient NRCMs after CHX treatment, and AKT protein in *ctrl*‐NRCMs degraded starting from 12 h after CHX treatment, while the degradation rate of AKT protein in LRP5‐deficient NRCMs accelerated compared with that in the *ctrl* group (Figure 5C). To detect the pathway by which LRP5 regulated the degradation of AKT protein, we then treated control and LRP5‐deficient NRCMs with hydroxychloroquine (HCQ, lysosomal inhibitor) or MG132 (a proteasome inhibitor). We found HCQ had no significant effect on the reduction of AKT expression induced by LRP5 deficiency, while MG132 could rescue the reduction of AKT expression (Figure 5D and E). In addition, considering that ubiquitination of AKT is a critical step in the proteasome‐mediated protein degradation pathway, we also examined this process. The results showed that LRP5 knockdown increased the ubiquitination of AKT protein (Figure 5E). These results indicated that LRP5 deficiency accelerated AKT degradation by promoting the proteasomal degradation pathway.

Furthermore, we also examined the effect of LRP5 deficiency on cell cycle gene expression. We found that LRP5 deficiency increased P21 expression, while the expression of P27, p‐P27, P53, P38 and p‐P38 was not changed (Figure 5F). These data suggested that LRP5 regulated cardiomyocyte proliferation by directly modulating the proteasomal degradation of AKT and then affecting the expression of P21.

## 4 DISCUSSION

We demonstrated a critical role of LRP5 in cardiomyocyte proliferation and neonatal heart regeneration. The expression of LRP5 was positively correlated with the proliferative ability of cardiomyocytes. LRP5 knockdown reduced the proliferation of cardiomyocytes. Moreover, the *Lrp5*‐CKO mice displayed impaired capacity of myocardial regeneration due to the inhibition of cardiomyocyte proliferation. Further studies revealed that LRP5 directly bound to AKT. LRP5 deficiency accelerated the protein degradation process of AKT through the proteasome pathway, thereby reducing cardiomyocyte proliferation.

This study revealed an uncovered cellular role of LRP5 in cardiomyocyte proliferation. The correlation of LRP5 expression with cardiomyocyte proliferation suggested that LRP5 was involved in cell cycle arrest in cardiomyocytes during the postnatal period. Knockdown of *Lrp5* demonstrated its inhibitory effect on cardiomyocyte proliferation, as evidenced by the DNA synthesis marker EdU and G2/mitosis marker pH3‐S10. Moreover, LRP5 overexpression promoted NRCM proliferation, further confirming the critical role of LRP5 in the cell cycle regulation of cardiomyocytes. In addition, enlarged cardiomyocytes were also observed in *Lrp5*‐CKO mice, suggesting an imbalance in homeostasis between cell cycling and cell growth caused by *Lrp5* deficiency. Deciphering the effect of LRP5 on the cell cycle re‐entry of cardiomyocytes in different growth periods might provide important insights into myocardial regeneration.

Endogenous regeneration after cardiac injury has been proposed as a novel approach to repairing myocardial damage.^7^, ^34^ Here, we demonstrated that *Lrp5* deficiency led to an incomplete heart regeneration response to AR surgery. In addition, cardiomyocyte proliferation and vascular density were examined in *Lrp5*‐CKO hearts after apex resection. The results indicated that impaired regeneration in the neonatal hearts of *Lrp5*‐CKO mice was caused by reduced cardiomyocyte proliferation. The identification of LRP5 in neonatal heart generation suggests that it is a potential target for the repair of myocardial injury. It has been reported that LRP5 knockout increases the infarct area after myocardial infarction in mice, suggesting a potential protective role of LRP5 in injured myocardium.^35^ However, whether LRP5 could promote adult cardiac repair by inducing cardiomyocyte proliferation needs to be explored in further work.

The AKT pathway is critically involved in the regulation of cardiomyocyte proliferation, cell growth and survival.^9^, ^32^, ^33^, ^36^, ^37^, ^38^ It has been shown that neuromodulin 1 (NRG1) and its specific tyrosine kinase receptor Erbb2/4 can promote cardiomyocyte proliferation by activating the AKT signalling pathway.^9^ In addition, the AKT pathway is essential for the regulation of cardiomyocyte proliferation mediated by YAP.^17^ Notably, the regulation of cardiomyocyte proliferation associated with AKT pathway mainly through the alteration of AKT phosphorylation rather than total AKT protein expression. In our study, LRP5 downregulation accelerated the proteasomal degradation of the total AKT protein, thereby promoting the expression of the cell cycle gene P21, providing another perspective on the mechanisms underlying cardiomyocyte proliferation mediated by the AKT pathway.

LRP5 is highly homologous with LRP6, which has similar biological roles in canonical Wnt signalling. Interestingly, our studies revealed their distinct roles in cardiac pathophysiology. We found that *Lrp6*‐CKO mice displayed a normal QT interval,^39^ while *Lrp5*‐CKO mice displayed a shortened QT interval.^31^ Recently, we reported that LRP6 acts as an RNA‐binding protein to regulate the mRNA degradation of *Ing5* and LRP6 deficiency promotes cardiomyocyte proliferation.^40^ Here, we revealed that LRP5 regulates the proteasomal degradation of AKT through directly binding to AKT protein. In addition, LRP5 deficiency inhibited cardiomyocyte proliferation. The findings of LRP5 and LRP6 in cardiomyocyte proliferation suggest their roles in homeostasis maintenance of the cell cycle and cardiac regeneration ability.

In conclusion, our study revealed that LRP5 was essential for cardiomyocyte proliferation and neonatal heart regeneration. LRP5 directly bound to AKT and regulated its protein degradation, thereby affecting P21 expression. The uncovering of LRP5 in neonatal heart regeneration may provide new insights into the repair of myocardial injury.

## CONFLICT OF INTEREST

The authors declare that they have no conflict of interest.

## AUTHOR CONTRIBUTION

**Huixing Zhou:**Data curation (lead); Formal analysis (equal); Investigation (equal); Methodology (equal); Visualization (lead); Writing – original draft (equal). **Fulei Zhang:** Data curation (equal); Formal analysis (lead); Investigation (lead); Methodology (equal); Writing – original draft (equal). **Yahan Wu:** Data curation (equal); Methodology (equal). **Hongyu Liu:** Investigation (equal); Methodology (equal). **Ran Duan:** Investigation (equal); Methodology (equal). **Yuanyuan Liu:** Investigation (equal). **Yan Wang:** Investigation (equal). **Xiaoyu He:** Investigation (equal). **Yuemei Zhang:** Investigation (equal). **Xiue Ma:** Investigation (equal). **Yi Guan:** Investigation (equal). **Yi Liu:** Investigation (equal). **Dandan Liang:** Conceptualization (equal); Funding acquisition (equal); Writing – review & editing (equal). **Liping Zhou:** Conceptualization (equal); Data curation (equal); Methodology (equal); Supervision (equal); Writing – original draft (lead); Writing – review & editing (equal). **Yi‐Han Chen:** Conceptualization (lead); Funding acquisition (lead); Project administration (lead); Resources (lead); Supervision (lead); Writing – review & editing (lead).

## Supporting information

Supplementary Material

## Data Availability

The data that support the findings of this study are available from the corresponding author upon reasonable request.
