# Supplementary material for: LRP5 regulates cardiomyocyte proliferation and neonatal heart regeneration by the AKT/P21 pathway
Source: J Cell Mol Med. 2022 Apr 16;26(10):2981–94. doi: 10.1111/jcmm.17311 (PMC9097834; doi:10.1111/jcmm.17311)
Supplement: Supplementary file 1 — Supplementary Material [file JCMM-26-2981-s001.docx]

**Supplementary Materials**

**Supplementary Figures**

**
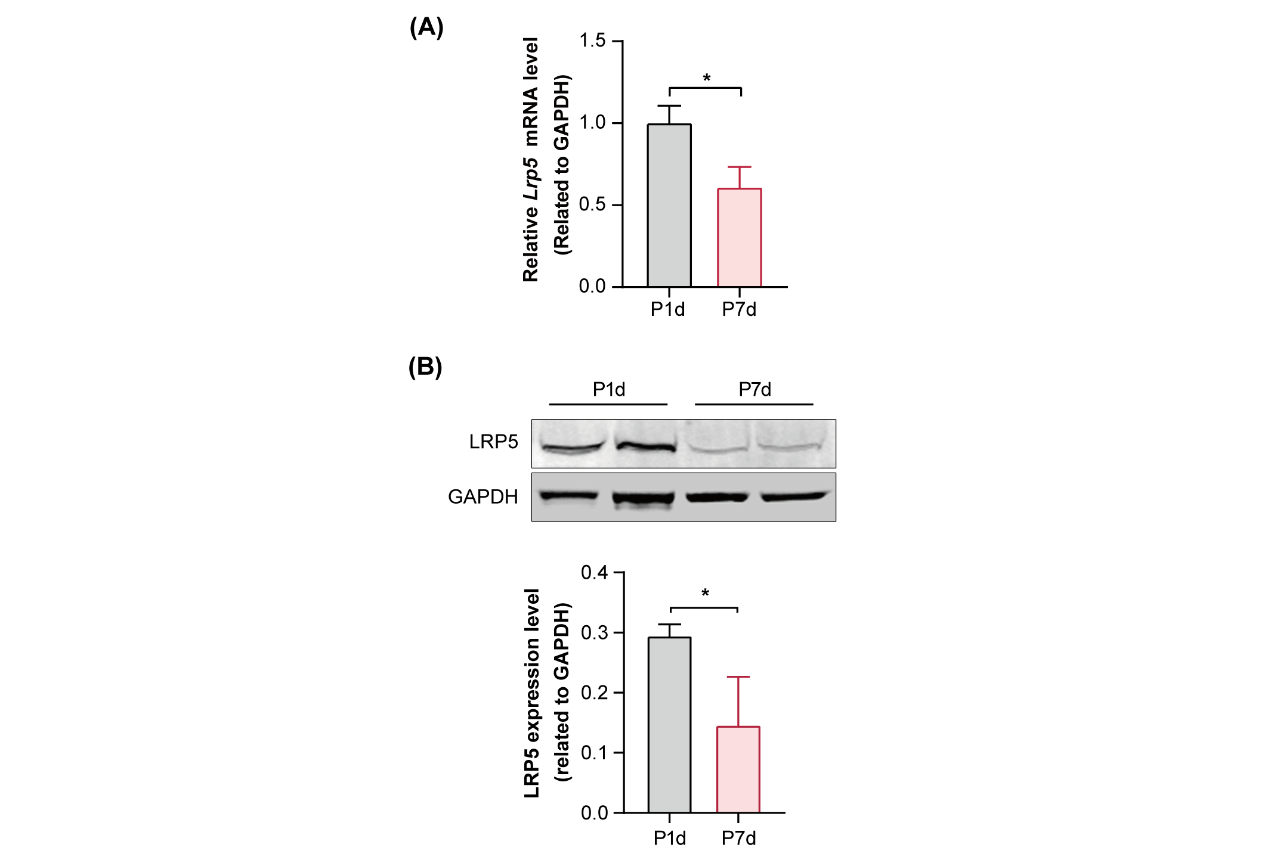
 Supplementary Figure 1. The expression of LRP5 in P1d and P7d mice cardiomyocytes (CMs).** (A) The qPCR assay shows the mRNA expression of *Lrp5* in P1d and P7d mice CMs. (B) Representative western blot bands and pooled data showing the expression of LRP5 is significantly reduced in neonatal mice cardiomyocytes (NMCMs) on P7d. Neonatal mice CMs were isolated from P1d and P7d mice hearts, respectively. n = 3 independent experiments. ***p* < 0.01. The data are presented as the means ± SD.


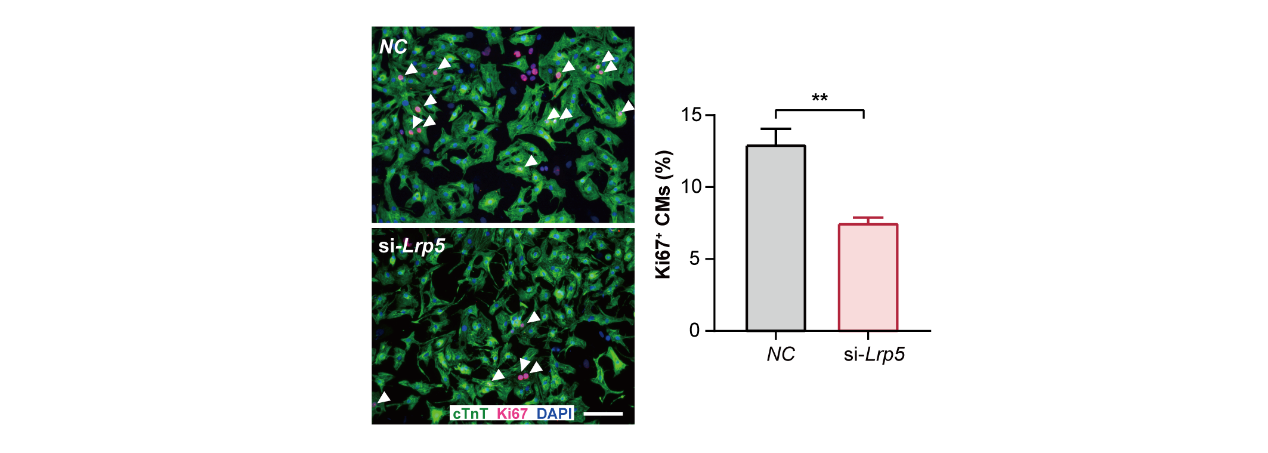


**Supplementary Figure 2. Evaluation of proliferative activity in NRCMs by Ki67 staining following LRP5 knockdown**. Representative images of NRCMs staining with Ki67 and cTnT in negative control (*NC*) or si-*Lrp5* group. White arrows indicate Ki67^+^cTnT^+^ cells. The column showing the percentage of Ki67 positive cardiomyocytes. Scale bar, 100 µm. Values are the average ± SD of three independent experiments. *p* values were calculated using the unpaired Student’s *t* test (***p* < 0.01).


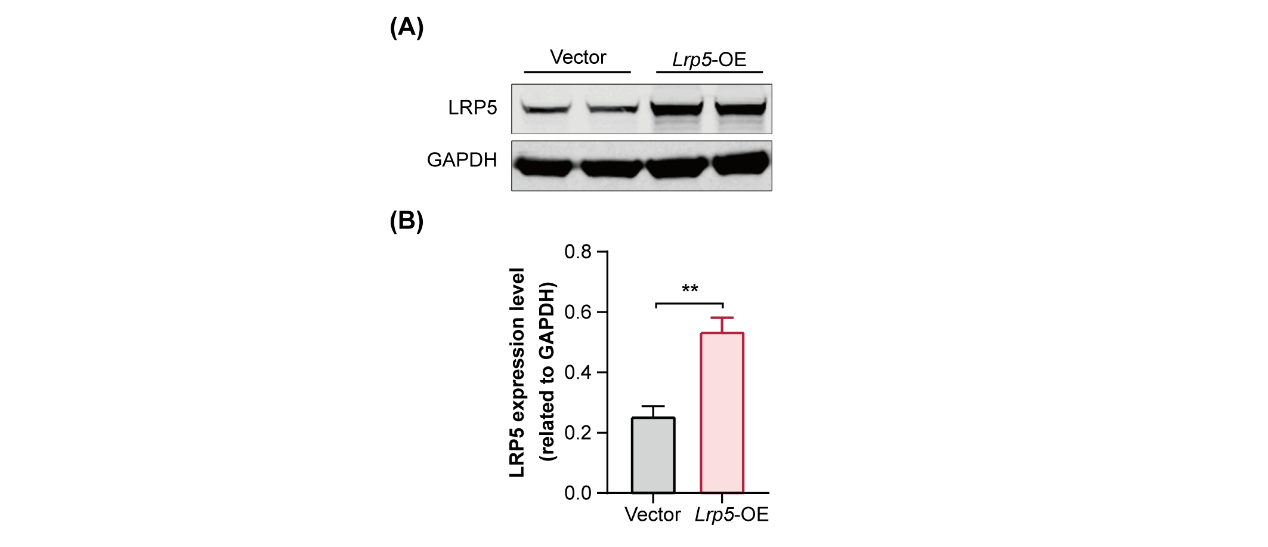


**Supplementary Figure 3. Western blot analysis showing LRP5-overexpressing plasmid efficiency in NRCMs.** Representative western blot bands (A) and quantification (B) showing the efficiency of the *Lrp5*-overexpressing plasmid (*Lrp5*-OE) in NRCMs. n = 3 independent experiments. The data are presented as the means ± SD. ***p* < 0.01.


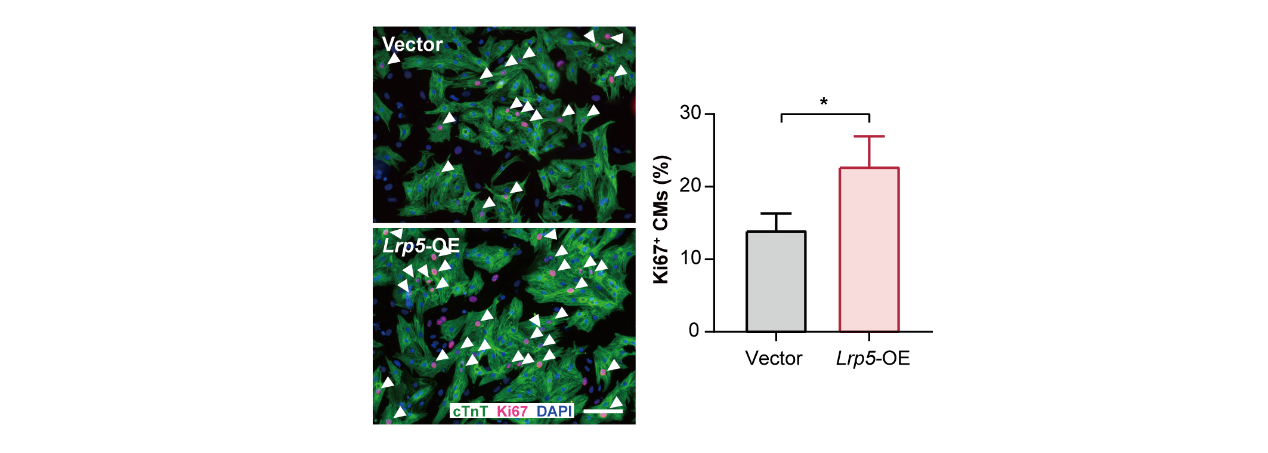
**Supplementary Figure 4. Evaluation of proliferative activity in NRCMs by Ki67 staining following LRP5 overexpression.** Representative images of NRCMs staining with Ki67 and cTnT in negative control (vector) or *Lrp5* overexpression (*Lrp5-*OE) group. White arrows indicate Ki67^+^cTnT^+^ cells. The column showing the percentage of Ki67 positive cardiomyocytes. Scale bar, 100 µm. Values are the average ± SD of three independent experiments. *p* values were calculated using the unpaired Student's *t* test (**p* < 0.05).


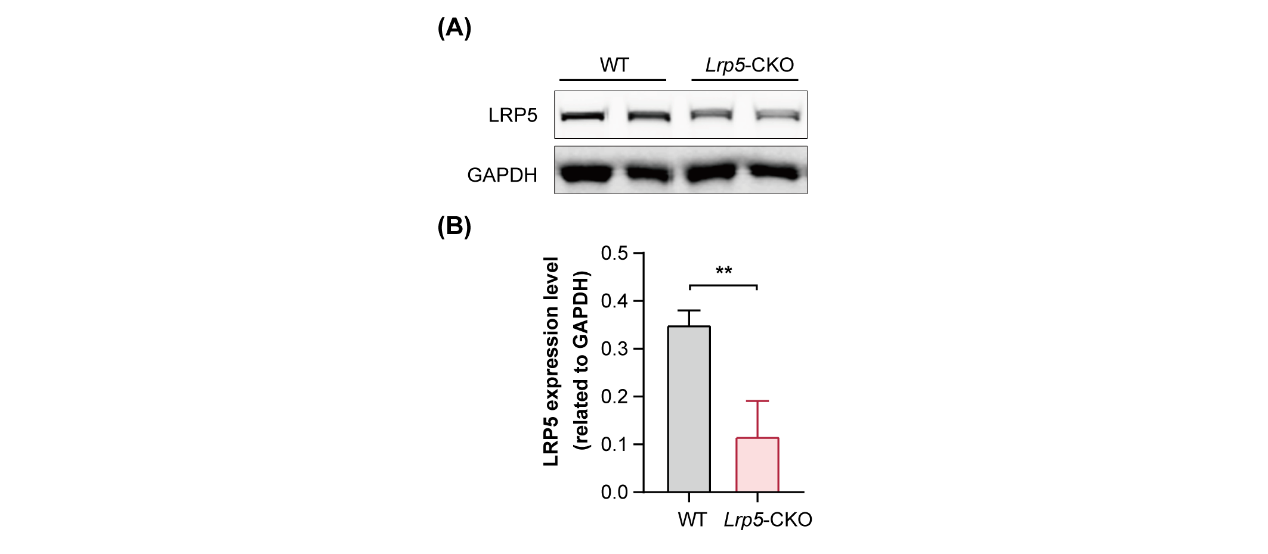
 **Supplementary Figure 5. The deletion efficiency of LRP5 in *Lrp5*-CKO hearts.** The neonatal mice (P0d) were injected with tamoxifen (40 ug) for 3 days, the total protein extracted from wild type (WT) and cardiac-specific knockout of *Lrp5* (*Lrp5*-CKO) mouse heart tissues for the examination of *Lrp5* deletion. Representative western blot bands (A) and pooled data (B) of relative LRP5 protein expression in P7d WT and *Lrp5*-CKO mice heart tissues. GAPDH was used as a loading control. n = 3 mice per group. ***p* < 0.01. The data are presented as the means ± SD.


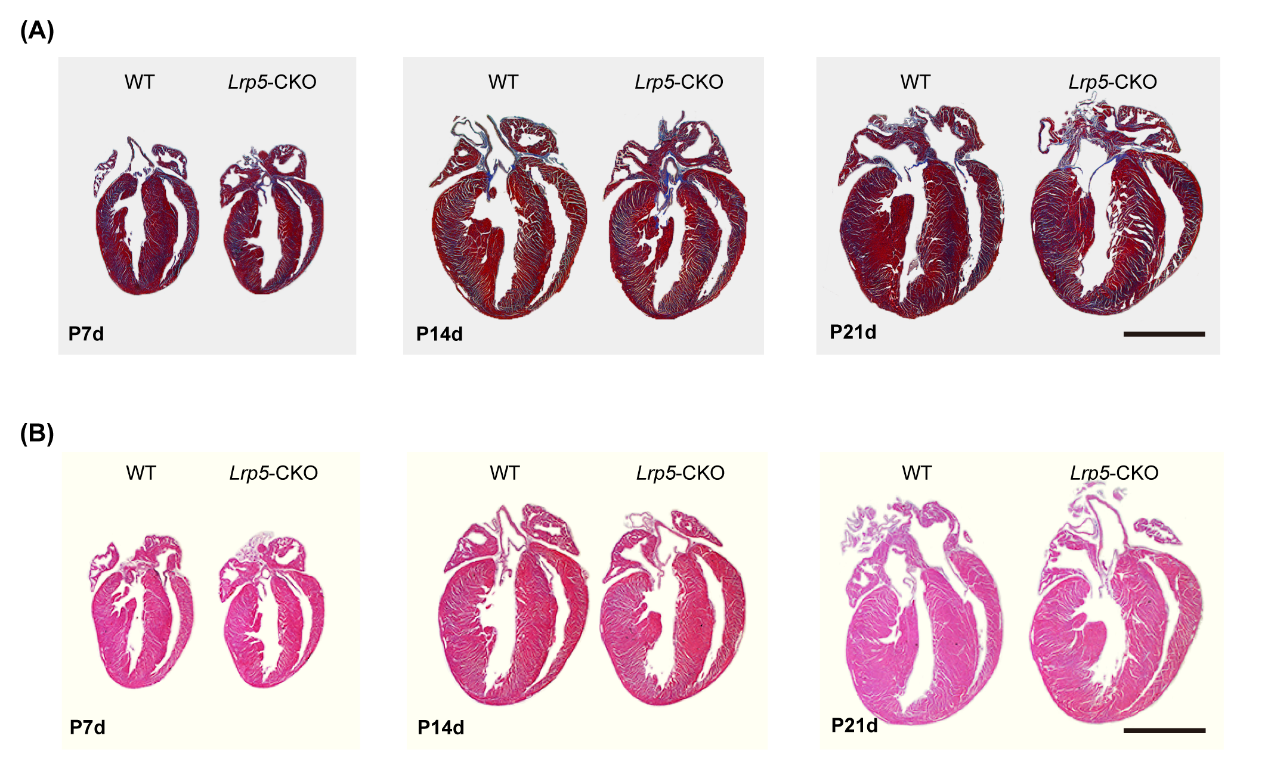


**Supplementary Figure 6. Cardiac fibrosis and morphology in WT and *Lrp5*-CKO mice.** (A) Masson’s trichrome staining of WT and *Lrp5*-CKO mouse hearts at P7d, P14d, and P21d. Scale bar, 2 mm. (B) H&E staining of WT and *Lrp5*-CKO mouse hearts at P7d, P14d, and P21d. Scale bar, 2 mm.


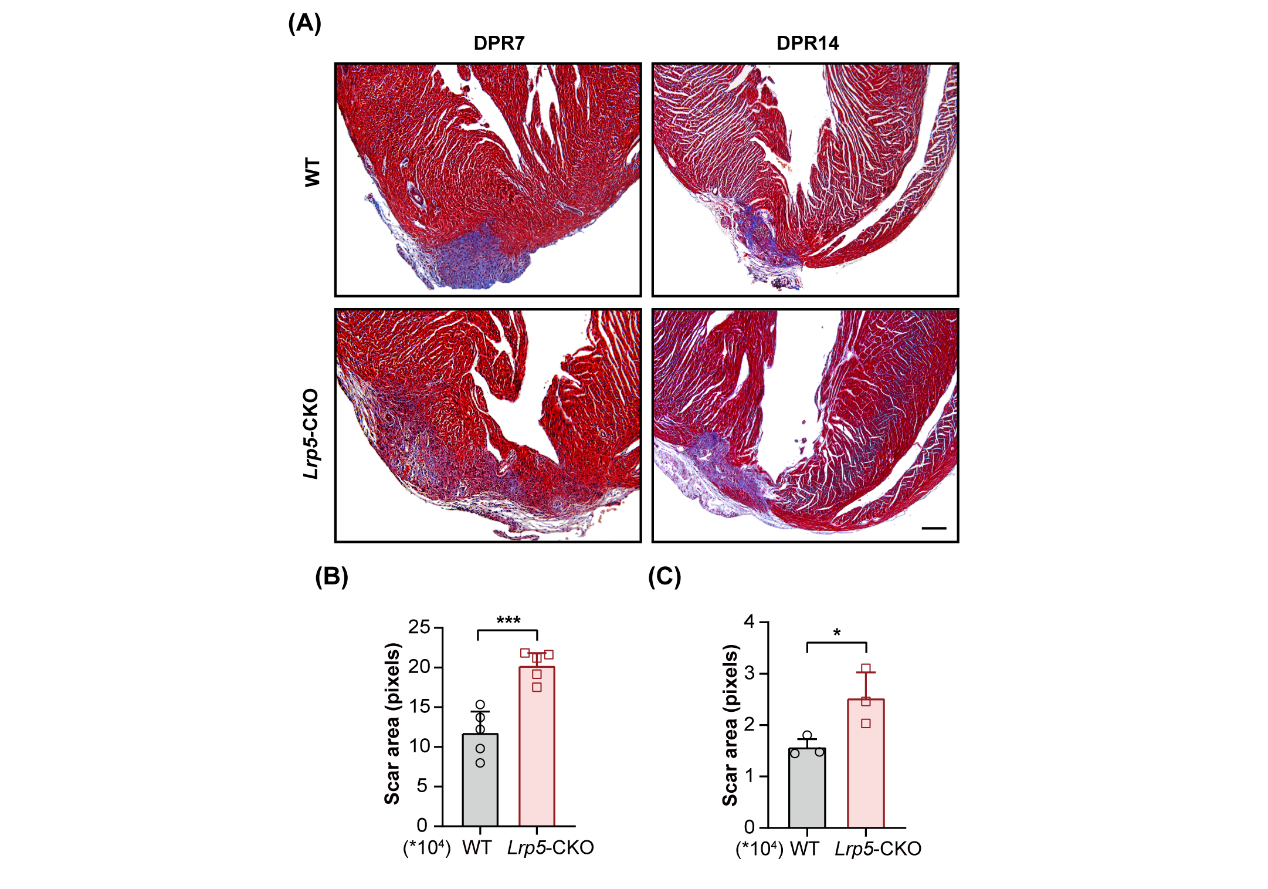


**Supplementary Figure 7. Scale size of WT and *Lrp5*-CKO mouse hearts at DPR7 and DPR14.** (A) Representative images of Masson’s trichrome staining of WT and *Lrp5*-CKO mouse hearts at DPR7 and DPR14. Scale bar, 100 µm. (B) Quantitation of scar size in WT and *Lrp5*-CKO mouse hearts at DPR7. n = 5 mice per group. (C) Quantitation of scar size in WT and *Lrp5*-CKO mouse hearts at DPR14. n = 3 mice per group. The data are presented as the means ± SD.**p* < 0.05, ****p* < 0.001.


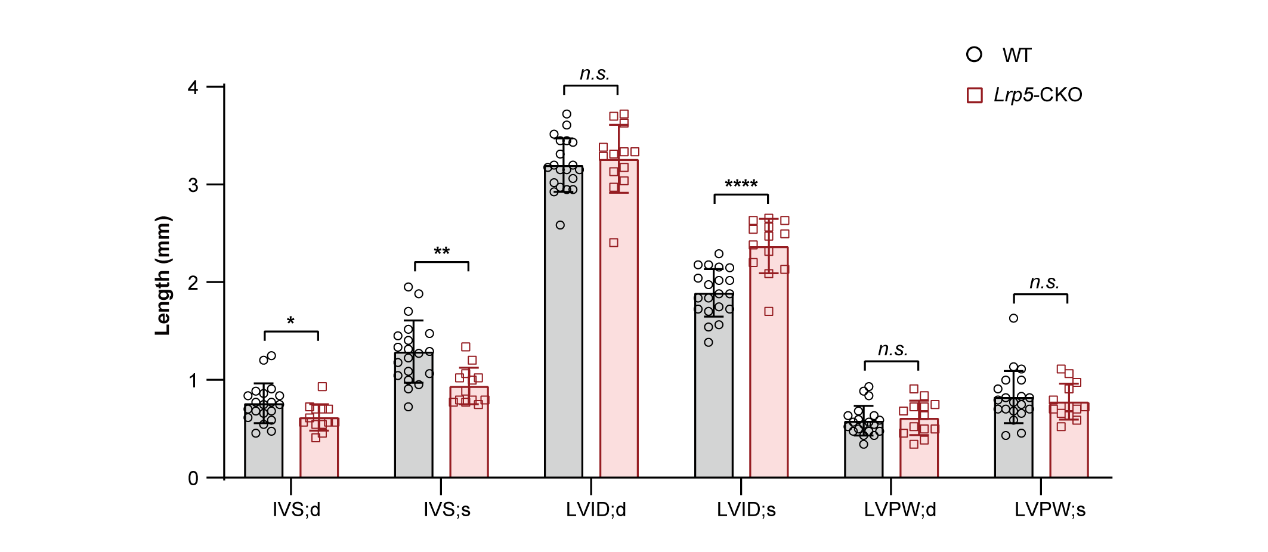


**Supplementary Figure 8. Two-dimensional echocardiographic measurements of WT and *Lrp5*-CKO mice at DPR21.** The diastolic interventricular septal thickness (IVS; d), systolic interventricular septal thickness (IVS; s), diastolic left ventricular diameter (LVID; d), systolic left ventricular diameter (LVID; s), diastolic left ventricular posterior wall thickness (LVPW; d), and systolic left ventricular posterior wall thickness (LVPW; s) between WT and *Lrp5*-CKO mice at DPR21. n = 13-20 mice per group. The data are presented as the means ± SD.**p* < 0.05, ***p* < 0.01, *****p* < 0.0001, *n.s.*, no significance.


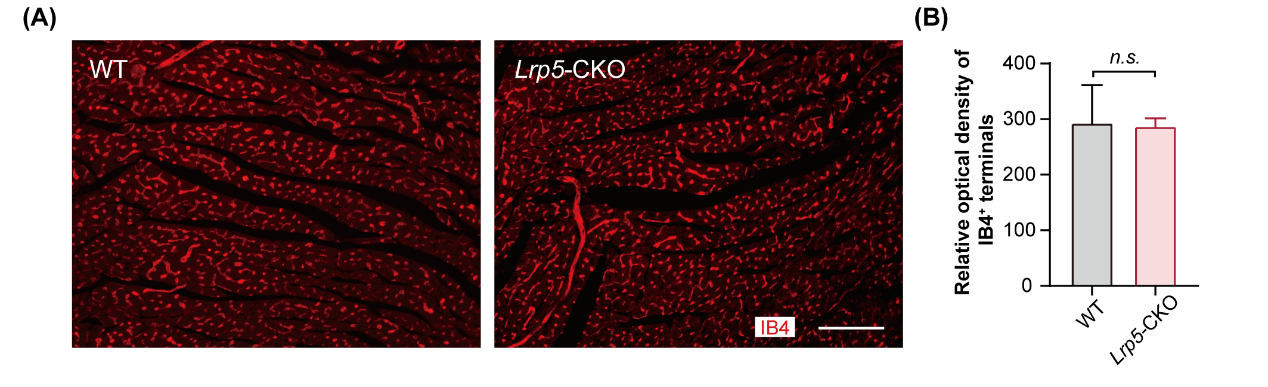


**Supplementary Figure 9. Angiogenesis in the apical sections of *Lrp5*-CKO hearts.**

(A) Immunofluorescence staining for isolectin B4 (IB4) in WT and *Lrp5*-CKO mice at DPR21. IB4 staining was used to examine for vessel density. (B) Quantification of capillary density 21 days after injury in the apical zone. Scale bar, 100 µm. n = 3 mice per group. The data are presented as the means ± SD. *n.s.*, no significance.


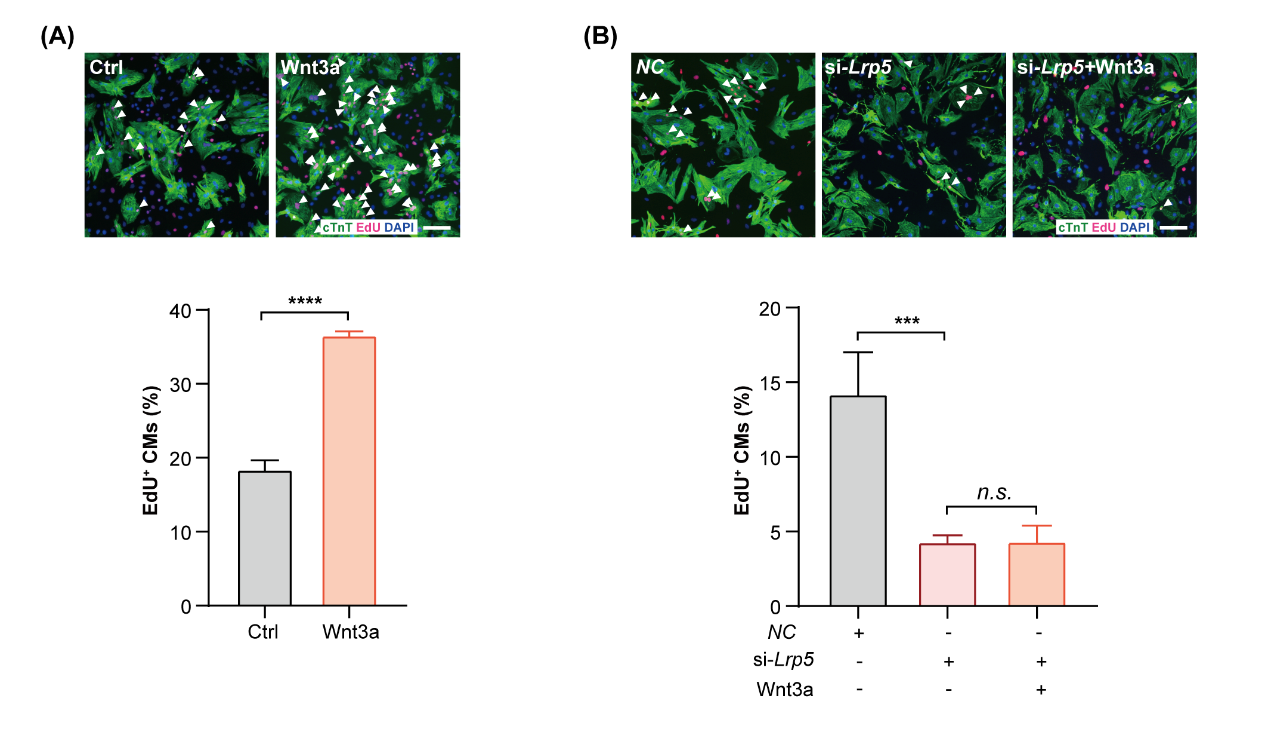


**Supplementary Figure 10. Wnt3a could not attenuate the decrease of cardiomyocyte proliferation caused by LRP5 deficiency.** (A) *Top,* representative images showing the EdU staining in NRCMs treated with Wnt3a or DMSO (Ctrl). White arrows indicate the EdU^+^cTnT^+^ cells. *Bottom*, column showing the quantification of EdU^+^cTnT^+^ cells. Scale bar, 100 μm. n = 3 independent experiments. The data are presented as the means ± SD. *****p* < 0.0001. (B) EdU assays showing that the decreased proliferation of cardiomyocytes induced by LRP5 deficiency could not be attenuated by Wnt3a. NRCMs were transfected with *Lrp5*-siRNA for 48 h, then treated with Wnt3a (0.1 mg/ml) for 24 h. *Top*, representative images showing EdU staining; *bottom*, quantification of EdU^+^cTNT^+^ cells. Scale bar, 100 µm. n = 4 independent experiments. The data are presented as the means ± SD. ****p* < 0.001, versus *NC* group, *n.s.*, not significant, versus *Lrp5*-siRNA treated group.


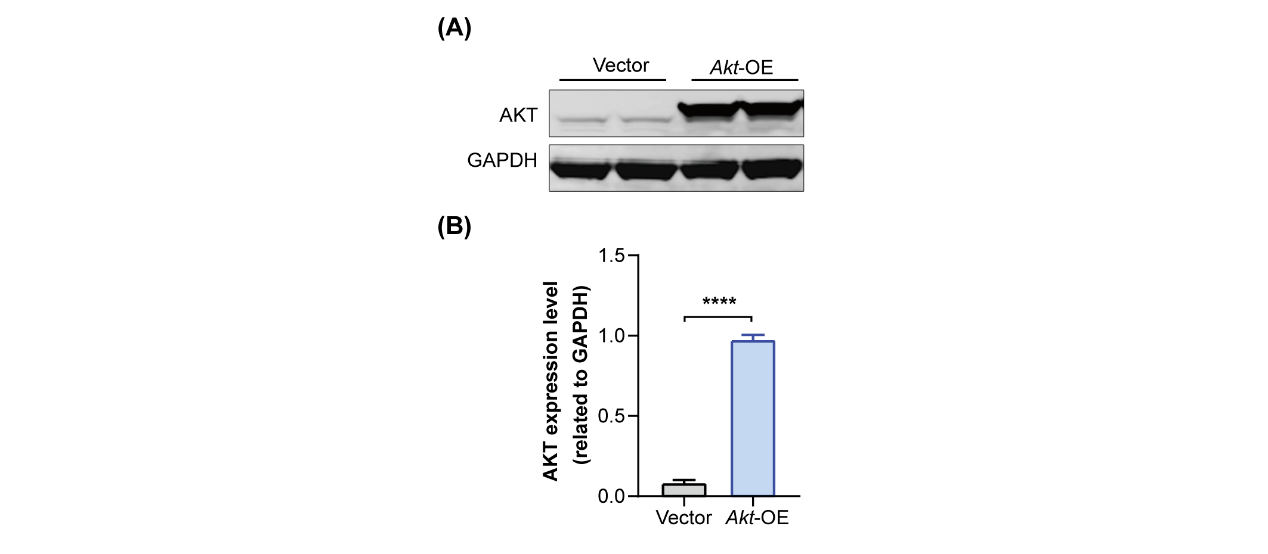


**Supplementary Figure 11. Efficiency of the AKT-overexpressing plasmid in NRCMs.**

Representative western blot bands **(**A**)** and quantification **(**B**)** showing the efficiency of the *Akt*-overexpressing plasmid (*Akt*-OE) in NRCMs. n = 3 independent experiments. The data are presented as the means ± SD. *****p* < 0.0001.


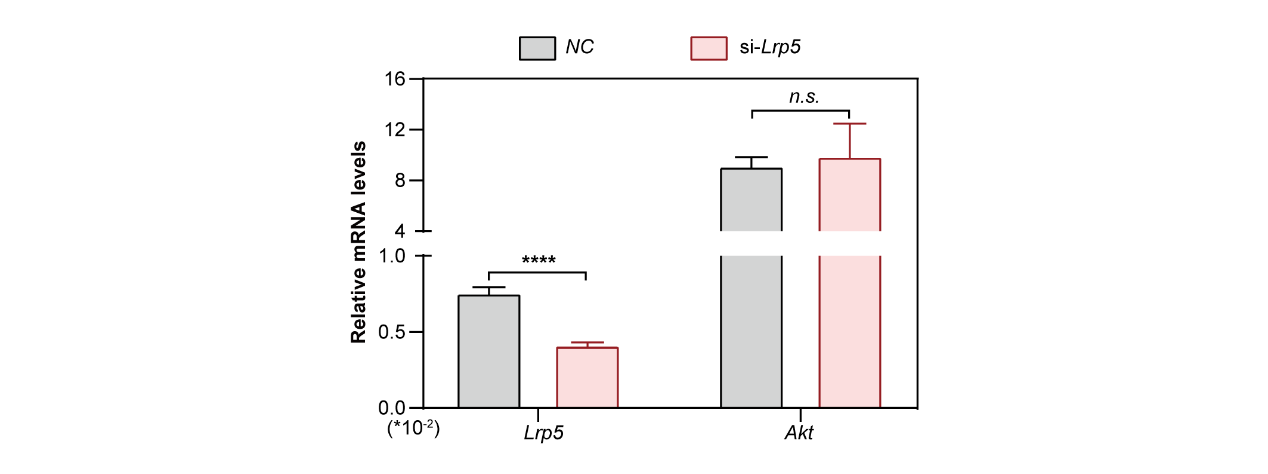


**Supplementary Figure 12. LRP5 downregulation did not affect *Akt* mRNA expression.** qPCR showing *Akt* mRNA expression after LRP5 deletion in NRCMs. n = 3 independent experiments. The data are presented as the means ± SD.*****p* < 0.0001, *n.s.*, no significance.


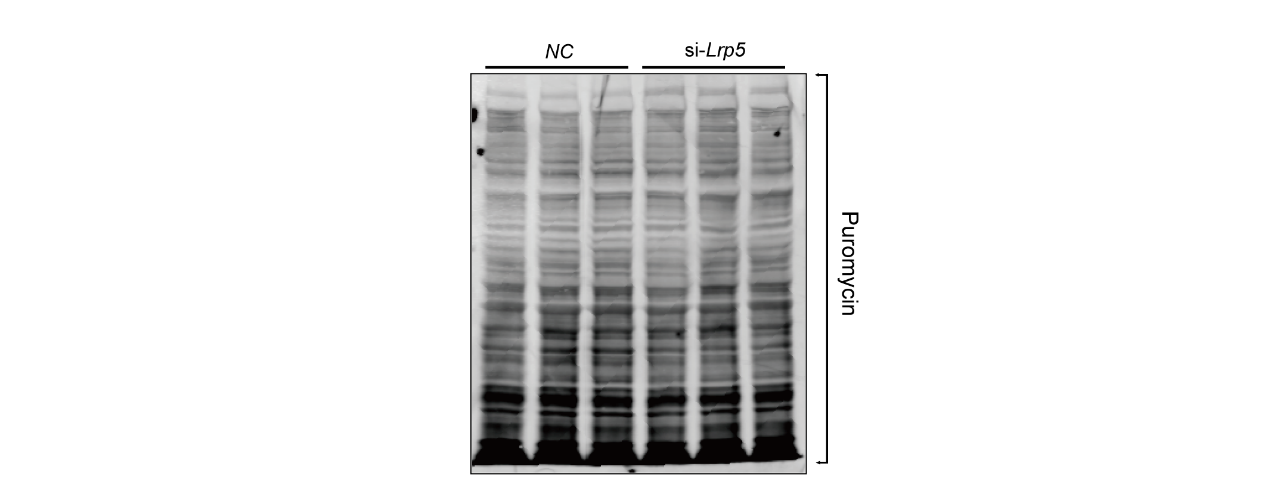


**Supplementary Figure 13. LRP5 deficiency did not affect the synthesis of nascent proteins.** Western blot showing the level of puromycin after LRP5 deletion in NRCMs. NRCMs were transfected with *NC*-siRNA or *Lrp5*-siRNA and were incubated with 5 µM puromycin for 1 hour before collection. Then the cells were lysed and analyzed by western blot using antibody of puromycin. n = 3 independent experiments.

**Supplementary Table 1. List of primers used for RT-PCR.**

| Gene | Forward Primer | Reverse Primer |
| --- | --- | --- |
| Mus-*Lrp5* | ACGTCCCGTAAGGTTCTCTTC | GCCAGTAAATGTCGGAGTCTAC |
| Mus-*GAPDH* | AGGTCGGTGTGAACGGATTTG | TGTAGACCATGTAGTTGAGGTCA |
| Rat-*Lrp5* | CCTGAGTGTGCTGACCAGAG | AGACAGCATCGCAGCGTAAA |
| Rat-*Akt* | ACCTCTGAGACCGACACCAG | AGGAGAACTGGGGAAAGTGC |
| Rat-*GAPDH* | ATGGGGAAGGTGAAGGTCG | GGGGTCATTGATGGCAACA |
